# Access to and use of sexual and reproductive health services provided by midwives among rural immigrant women in Spain: midwives’ perspectives

**DOI:** 10.3402/gha.v6i0.22645

**Published:** 2013-11-08

**Authors:** Laura Otero-Garcia, Isabel Goicolea, Montserrat Gea-Sánchez, Belen Sanz-Barbero

**Affiliations:** 1 National School of Public Health, Institute of Health “Carlos III”, Spain; 2 GESEC, Lleida University, Lleida, Spain; 3 Epidemiology and Global Health Unit, Department of Public Health and Clinical Medicine, Umeå University, Umeå, Sweden; 4 Faculty of Nursing, Lleida University, Lleida, Spain; 5 Ciber of Epidemiology and Public Health (CIBERESP), Spain

**Keywords:** gender, health services accessibility, immigrants, midwives, primary health care, qualitative research, rural population, sexual and reproductive health, utilization, women's health

## Abstract

**Background:**

There is insufficient information regarding access and participation of immigrant women in Spain in sexual and reproductive health programs. Recent studies show their lower participation rate in gynecological cancer screening programs; however, little is known about the participation in other sexual and reproductive health programs by immigrant women living in rural areas with high population dispersion.

**Objectives:**

The objective of this study is to explore the perceptions of midwives who provide these services regarding immigrant women's access and participation in sexual and reproductive health programs offered in a rural area.

**Design:**

A qualitative study was performed, within a larger ethnographic study about rural primary care, with data collection based on in-depth interviews and field notes. Participants were the midwives in primary care serving 13 rural basic health zones (BHZ) of Segovia, a region of Spain with high population dispersion. An interview script was designed to collect information about midwives’ perceptions on immigrant women's access to and use of the healthcare services that they provide. Interviews were recorded and transcribed with participant informed consent. Data were analyzed based on the qualitative content analysis approach and triangulation of results with fieldwork notes.

**Results:**

Midwives perceive that immigrants in general, and immigrant women in particular, underuse family planning services. This underutilization is associated with cultural differences and gender inequality. They also believe that the number of voluntary pregnancy interruptions among immigrant women is elevated and identify childbearing and childrearing-related tasks and the language barrier as obstacles to immigrant women accessing the available prenatal and postnatal healthcare services.

**Conclusions:**

Immigrant women's underutilization of midwifery services may be linked to the greater number of unintended pregnancies, pregnancy terminations, and the delay in the first prenatal visit, as discerned by midwives. Future research should involve samples of immigrant women themselves, to provide a deeper understanding of the current knowledge, attitudes, and practices of the immigrant population regarding reproductive and sexual health to provide better health services.

Research on migrant access and utilization of health services has proliferated in the last decades due to the growth of immigrant population settling into Europe (1). Results point to a lower utilization rate of services by immigrants compared to native-born, although figures display great heterogeneity due to the diversity in host country, place of origin of the immigrants, and the specific healthcare services examined (2–4).

Several authors have associated this lower utilization rate to the commonly healthier status of the immigrant population, compared to the native-born, as well as to the existence of barriers to access and use of health services (5, 6). These barriers gain more relevance in rural areas with high population dispersion where the distance to reach health facilities alone limits access and use of services (4).

The fertility rate rebound observed in Spain from 2006 until today (1.38 children/woman) is a reflection of the recent substantial increase in immigration rates (7–11) Immigrant women residing in Spain exhibit different sexual and reproductive health patterns from native-born Spaniards: greater fertility, lower age at first birth, greater rates of premature births and more births to infants with low birth weight (7, 8, 12, 13), as well as a higher proportion of voluntary terminations of pregnancies (VTP) (7, 13).

Despite having quantitative data on these differences, information on access to and participation in sexual and reproductive health programs by immigrant women in Spain is very scarce. Recent studies show a lower rate of participation in gynecological cancer screening programs among these women (14), but little is known about participation in sexual and reproductive health programs, especially among immigrant women living in rural areas. Given this gap in the literature, the objective of our study is to examine the perceptions of the professionals providing these services, the midwives, on the topic. The actual implementation of public health programs is strongly dependent on service providers, who may observe, adapt or completely ignore the programs (15). Their attitudes and practices can enhance or hinder women's access to and use of services. With this purpose, midwives were interviewed regarding access and participation in sexual and reproductive health programs offered in an area with high population dispersion.

## Methods

The geographical context of this study is the rural area of Segovia, a province in one of Spain's Autonomous Regions, known as Castile and Leon. This is the largest region in the country and the one with the lowest population density (27 inhabitants/km^2^).

About 64% of the population of Segovia (104,895 inhabitants) (16) lives in rural areas composed of 208 municipalities and 17 local authorities. The area has a high population dispersion (28% of the municipalities report fewer than 100 inhabitants) and a low population density (23 inhabitants/km^2^) (16). Segovia is experiencing population loss and population aging: 21% of the population is over 65 years of age (16), though this figure is only 12.5% in the receiving immigrant population (16).

Segovia's health area is divided into 16 basic health zones (BHZ), of which three are urban and 13 are rural. Each BHZ has a primary care center where the midwife's office is located. Seven midwives cover the 13 rural BHZ, where one midwife may cover between one and three BHZs.

Based on an ethnographic design, this study focused on primary care and healthcare processes in the rural environment (17). The main author between February 2008 and November 2009 performed fieldwork, including interviews. The qualitative study design included in-depth interviews with seven midwives serving in rural areas. The interview script was designed to collect information about midwives’ views about access to and use of midwifery services (18).

After informing the participants about the goals of the study and guaranteeing confidentiality, their consent was secured for participating and recording the interviews. An external expert transcribed all the interviews. The transcriptions of these recordings were analyzed by two of the authors based on the qualitative content analysis approach (19).

The data analysis was developed by two of the authors who first read the transcripts. Then, they imported the text into Open Code software to manage the coding process (20). First, parts of the text relating to the research question were identified (meaning units) and short summarized versions of them were developed (condensed meaning units). From the condensed meaning units, codes were then produced. The codes were grouped together into two emerging categories, which relate to the manifest content of the text. Finally, a theme emerged that cut across both categories and refers to the latent content. During the analysis, consensus about the results was reached among three of the authors. To support this analysis, they also used notes collected during the fieldwork.

In this article, the word *midwife* includes both male and female midwives. The term *immigrant women* indicates women of foreign origin, whom midwives sometimes refer to according to their country of origin as *Moroccan*, *Bulgarian*, or *Rumanian*.

## Ethics approval

The research protocol was approved by the Ethics Committee of the Health Institute Carlos III (Spain). This study was funded by National Health Funding Research-project PI 080306.

## Results

Results regarding rural midwives’ perceptions are organized into two categories: (1) place of origin and socio-economic situation (being an economic immigrant) and gender (being a woman) affects family planning; (2) there are access barriers to and underutilization of available prenatal and postnatal healthcare services by immigrant women. The theme that combines both of these categories is: midwives’ perceptions of underutilization of sexual and reproductive services by immigrant women.

### Culture as the source of difficulties with family planning for immigrant women

According to rural midwives, immigrant populations residing in the rural areas of Segovia hardly engage in any family planning, which midwives interpret as a consequence of ‘cultural differences’.

In this area, immigrants are mostly Bulgarian and Romanian. They don't use contraception […] these are people who you explain things to and maybe you get them to agree with the analysis, or something, and then, they don't do it. They won't take the pill, they won't get an IUD, they don't use condoms … and I think it's cultural. (Midwife 4)

I think that immigrant women do not have the issue of prevention and family planning incorporated. It is true that until they are sick many do not come to me. It can be cultural. (Midwife 3)

When asked to elaborate on what they meant by *cultural differences*, midwives explained that it was the men who decided whether or not to use family planning methods, since they often held negative attitudes regarding taking any action around contraception and whether it is for the woman or themselves. Thus, the decisions of men prevail over women's decisions.

Many are influenced by their husbands, their partners. They tell you that their husbands are in control. Then, you feel very frustrated. I ask them-What about an IUD? No, my husband doesn't want me to get an IUD. –What about the pill?- No, my husband doesn't want me to take anything. –Well, then tell your husband to use a condom- But he doesn't want to use a condom either. (Midwife 2)

Immigrant women know about contraceptive methods because I explain to them when they come to see me, but for them it is easier not to use anything. Their partners don't even want to use a condom. (Midwife 5)

Less often, midwives perceived that immigrant women used family planning methods but without their partner's knowledge. This shows the decisions of men are above the decisions of women, gender inequality is evident in this case.

A few of my patients took the pill without their partners’ knowledge, but those are the exceptions. (Midwife 7)

Some women tell me they want to plan their families, but they do not want their partners to know, because they (the partners) do not want to plan. (Midwife 6)

Midwives asserted that if a few immigrant women and their partners used family planning methods, they would serve as an example within their close social circle.

If one started to use it they would encourage others, because here in Spain, 40 years ago no one took the pill and if anyone did, they were told: ‘Oh no, that′s really bad for you, it causes cancer, you grow hair, you gain weight.’ Whatever their friend tells them always works better than anything that I tell them. (Midwife 1)

When an immigrant woman starts to come to the office to plan then her sisters come, then her sistersin- law . . .. (Midwife 5)

When faced with an unintended pregnancy VTP is one of the options considered by immigrant women. Midwives reported that VTPs are more common among women from Bulgaria and Romania as a consequence of the family planning policies in these countries, which are based on easy accessibility to VTP. In this way, midwives made references to the social, cultural, educational differences in immigrant women's notion of VTP, pregnancy, and family planning.

I have realized that Bulgarian and Rumanian women use abortion as a method of family planning. (Midwife 2)

There are countries like Bulgaria and Romania, where family planning was based on voluntary abortion. Abortion was promoted, and I believe that makes it less important to go for a visit to check there are no problems with the pregnancy, similarly, they don't have the same take on contraception that we do. (Midwife 3)

Some of the midwives pointed out that immigrant women did not always go to specialized centers to carry out these procedures (VTP), among others, but instead, some searched for alternate strategies despite the risks.

There is a medication, not sure they all know about it, but I'm convinced they get it. It's sold on the internet. I visited the site once and they give you addresses and phone numbers. I bet that they are selling it. It's used in the hospital environment for the stomach, but, of course, you're in a controlled environment. There's a risk of hemorrhaging, they may start bleeding and, just imagine, they think they've got it all out but a portion stays in, and can cause an infection. I've been asked for it, and the ones that ask for it are foreign women. Not Spanish women, which doesn't mean they don't know about it. (Midwife 3)

Midwives explained that these VTPs were performed either in Spain or in the immigrant women's country of origin, and that they (either themselves or their partners) bore the expenses.

There are Bulgarian women who go to Bulgaria for abortions. (Midwife 1)

Sometimes they abort here. Others go to their own countries, because, it's probably cheaper in their own country. Of course, here they might not be eligible for a legal abortion and they have to go to private clinics. That is why I think it's cheaper for them to go to Bulgaria. (Midwife 7)

Finally, midwives perceive that most of the teenage pregnancies occur among immigrant women.

I do not see many teenage pregnancies, but the few I see if there are more percentage in immigrant women. (Midwife 1)

Most of the teenage pregnancies we see are Romanian women from Rumania. (Midwife 4)

### Rural midwives perceived difficulties of access and use of prenatal and postpartum services among immigrant women

Midwives perceive that immigrant women make use of midwifery services mainly during pregnancies.

The only time when you really see a much higher proportion of immigrant women is during pregnancy. You don't see them during menopause, nor for contraception, but you see a few in Pap Smear and cervical cancer prevention programs, however during pregnancy is when you see them most. (Midwife 4)

For instance, I see Moroccan women in my office during their pregnancy and for post natal consultations, but I see them a lot less for pap smears. (Midwife 7)

However, midwives detect an underutilization of prenatal visits, which translates into a delayed first prenatal visit.

When Spanish women know they're pregnant they have the habit of going to their doctor or to the nurse, or the midwife, but they go to the health center. Immigrant women sometimes leave it longer. (Midwife 5)

There are women, especially Moroccans, who leave it longer to come. That's my experience. I think that if they come from a place where healthcare is not as accessible, then they are not used to going for medical care, and miss the usual first prenatal visit. (Midwife 6)

Regarding the program offering maternal education classes, midwives perceive that immigrant women use this program to a lesser extent than native-born women. Some of them compared immigrant women to women of Romany ethnicity.

About childbirth preparation group, sometimes I get that some Bulgarian or Moroccan women come. It is difficult to grasp them for activities like that. (Midwife 6)

They hardly come to the childbirth courses I offer, and if they attend one class, then they drop out. I'm not sure whether it is because it's silly, or because they can't follow it. You talk to them and then ask: -Do you understand? - and they answer affirmatively, but … It's the same thing with the gypsies, they don't come either. Maybe they think it's useless information, or they have other children to look after, or they have other things that prevent them attending … (Midwife 4)

Once again, midwives explain away this underutilization of their services based on ‘cultural differences’ regarding prenatal care. Some report that immigrant women think of pregnancy as a natural process which requires little supervision. Additionally, some midwives link this idea of immigrant women exhibiting an underdeveloped preventative culture with the fact that the immigrant population living in rural areas has a low socio-economic level. Other midwives talk about how women, in particular Moroccan women, do not attend these group activities because their husbands do not allow them.

Moroccan women relate to their children, with her husband, and very little with the rest of the people. I think we have a hard time doing group activities. I think they have restrictions by such husbands to attend childbirth preparation classes. In their culture the woman is in the private sphere. (Midwife 1)

It could be that for them pregnancy is not such a big deal … I don't mean they don't think it's important, but that they don't see the need for so much vigilance; it's something natural, and nothing will go wrong. In their country of origin they do go to be seen, I mean that they do follow the prenatal care. In Romania and Bulgaria for example, they do go as after all those countries are not so underdeveloped. As many people point out, the issue is that this type of immigrant is not their country's average citizen, but come from a lower social background; therefore culturally, prevention and care are lower. (Midwife 5)

Midwives explain that immigrant women sometimes skip scheduled appointments with them as well as with obstetricians in specialized care, going without some of the diagnostic tests in the prenatal protocol.

Immigrant are less reliable with their appointments, often they don't turn up, then they arrive without an appointment expecting to see you whenever it suits them, and things can get a bit chaotic. Of course there are all sorts of people, but you do see this more often with immigrants. (Midwife 4)

They are not as reliable when it comes to appointments; they are less likely to show up. Then they come with no appointment to be seen when it suits them, and this creates a degree of chaos. Many people do it but it is more common among immigrants, and especially within the Bulgarian population. (Midwife 7)

Some of the midwives point out that these sets of behaviors distinguish immigrant women from native-born women, except in those cases when native-born women live in socially dysfunctional situations.

Access to healthcare is relatively easy. If they don't go it's because they don't want to, because as sometimes happens with these women (immigrants) they don't even show up for blood test and miss hospital appointments … It's not all of them, but you don't see Spaniards doing that, and if you do, it's usually an isolated case with a family with issues. […] If you have six such cases per year, five are foreigners and one is not. (Midwife 2)

Finally, midwives also refer to language limitations as an access barrier for women from non-Spanish-speaking countries. Language limitation also results in these women's partners or their own children assisting with any communication with health professionals.

We get many from Morocco, the majority. We always give them, books about pregnancy and all that for them to read, although we mostly communicate with the husbands who know more, are more up-to-date, or with the kids, who speak very well. (Midwife 1)

Today I started a childbirth preparation group which should have like ten women, some of them immigrants. […] It is difficult to grasp for group activities for different reasons. One is the difficulty with the language, the language barrier. (Midwife 6)

## Discussion

The reproductive patterns described by the rural midwives in our study reflect official figures (7, 8). Data published by INE (Spanish acronym for the Spanish National Institute of Statistics) support the perceived high rates of VTPs that midwives believe their patients endure. These data show that in 2010, 41.7% of all VTP were performed on immigrant women (21), which is a very high percentage considering that in 2010 only 13.4% of women residing in Spain were immigrants (16).

In addition, the number of VTPs recorded among these women may be underestimated since, as the midwives indicated, some of these procedures are performed in the immigrant's country of origin, or in Spain but outside the public healthcare system. Finally, midwives argued that women from Eastern Europe choose VTP because this was a common practice in their countries of origin, encouraged by the legislation originated in those popular democracies (22).

Considering that 31.9% of adolescent pregnancies occur among immigrant women (23); that immigrant women have higher rates of VTP and higher fertility rates than autochthonous women (1.61 births per immigrant woman vs. 1.33 children per Spanish-born woman); that birth rates are also higher among immigrant women (17 immigrant mothers per 1,000 inhabitants vs. 9.5 Spanish-born mothers), we can say that there are family planning deficiencies among immigrant women.

According to the midwives the underutilization of family planning services, also described in relation to the cervical cancer prevention program elsewhere (2, 3, 14) is, again, likely related to cultural differences, the main barrier being the male partner's opposition, due to gender inequality (24), and to certain family planning practices.

It is worth noting that rural midwives do not think that the distance from the immigrants' municipality of residence to the rural health center is a barrier when seeking midwifery services. The fact that this potential barrier, observed and documented in previous studies (4, 25), fails to be perceived as an obstacle may reflect the existence of the communication difficulties between midwives and immigrant women. It is also possible that the women who do go to the midwife's office are those with no access barriers or those who managed to overcome them. However, the fact that midwives fail to perceive certain barriers to access and use of healthcare services already identified by different studies carried out in the same geographic environment (14, 25) may indicate a ‘blaming the victim’ attitude toward immigrant women. This viewpoint could prevent the launching of strategies aimed at improving access as well as utilization.

In summary, the midwives in our study equated the pattern of delayed access and underutilization of care often found in immigrant women with that of women of Romany ethnicity. That is, they identified immigrant populations with other groups at risk for social exclusion, as reported in a recent study (14). Furthermore, the association that these professionals made between low socio-economic level characteristic of the immigrant population and an underdeveloped preventative culture (these last one originated by the fact that they proceeded from low-income countries with limited preventative programs) has also been reported in quantitative studies as an association between lower socio-economic level and a lower utilization of preventative services (8, 26).

Regarding the underuse of maternal education programs, midwives emphasized the communication difficulties, specifically the language barrier, as an obstacle to access and use of such programs, as previously described by other authors (27, 28). They also identified the burden of childcare and childrearing, mostly the woman's responsibility in these immigrant populations, as a potential access barrier to prenatal care and services. This finding may be an indicator of how immigrant women's health is negatively impacted by the absence of the support received from traditional family and social network (29, 30).

Finally, it is important to comment that while this study provides evidence about midwifes’ perceptions on access to and use of midwifery services, it does not include the perceptions of the immigrant women users of these services. It is important to underline that research about immigrant women's perceptions would provide key insights into areas glossed over under culture by the midwives, and it could be an interesting area for further research.

## Conclusions

This study revealed perceptions of an underutilization of midwifery care among immigrant populations residing in the rural area of Segovia. According to the midwives working in rural primary care, this underuse results in unintended pregnancies, possible VTPs, and in delayed prenatal care. Further research is needed to gain a deeper understanding of the current knowledge, attitudes, and practices of the immigrant population, both men and women, regarding family planning, voluntary pregnancy interruption, and prenatal care to better match the supply and demand. Therefore, it is also necessary as appropriate delivery of reproductive and sexual health services for the immigrant population residing in rural areas of Segovia.
